# Probable Congenital Transmission of Reticuloendotheliosis Virus Caused by Vaccination with Contaminated Vaccines

**DOI:** 10.1371/journal.pone.0043422

**Published:** 2012-08-17

**Authors:** Kai Wei, Zhenhong Sun, Shufen Zhu, Wenlong Guo, Pengcheng Sheng, Zunmin Wang, Changliang Zhao, Qingyou Zhao, Ruiliang Zhu

**Affiliations:** Key Laboratory of Biological Product, College of Animal Science and Technology, Shandong Agricultural University, Taian, Shandong, People’s Republic of China; Commissariat a l’Energie Atomique(cea), France

## Abstract

Contaminated vaccine is one unexpected and potential origin of virus infection. In order to investigate the most likely cause of disease in a broiler breeder company of Shandong Province, all 17 batches of live-virus vaccines used in the affected flocks and 478 tissue samples were tested by dot-blot hybridization, nested PCR, and IFA. The results suggested the outbreak of disease was most probably due to the vaccination of REV-contaminated MD-CVI988/Rispens vaccines and ND-LaSota+IB-H120 vaccines. Furthermore, the REV was probably transmitted to the commercial chickens through congenital transmission.

## Introduction

The avian reticuloendotheliosis virus (REV), classified as a gamma-retrovirus, causes runting, immunosuppression, and lymphoma in a variety of avian hosts including chicken, turkey, duck, goose, pheasant, and peafowl [1], [2]. In recent years, immunosuppressive viruses represented by REV have been prevailing in China. Cheng et al. [3] performed serosurvey on broiler breeders in China during the period from September 2008 to November 2009, and discovered the antibody positive rate of REV was 42.6%. Yue et al. [4] detected the positive rate of REV was 59.0% among chickens of Sichuan Province by real-time polymerase chain reaction (PCR) in 2010. Because the outbreak of reticuloendotheliosis usually occurs at about 80 days of age in chickens, and REV often infects together with Marek’s disease virus (MDV) and avian leukemia virus (ALV) [2], [3], the vaccine manufacturers and the chicken keepers tend to neglect the detection and precaution against REV, which provides opportunities for the spread of REV.

In September 2010, three flocks (Flocks 2, 3, and 5; 25–30 weeks old) of a broiler breeder company in Shandong Province of China suffered emaciation and sporadic death with the death rate of around 0.8% in a week. Seven percent of the dead chickens showed the symptoms of visceral lymphomas. The egg production and hatchability were both lower than those of other normal flocks, and the death rate of embryos bred by the three broiler breeder flocks reached 2% after 19 days of hatching. Further investigation revealed that commercial chickens bred by the three broiler breeder flocks suffered poor and irregular growth, and demonstrated a poor immunological response to vaccination with Newcastle disease vaccines and avian influenza vaccines. Further, the 30 days old commercial chickens had a livability of about 93%. All of these respects corresponded to the typical characteristics of REV-infection. Case investigation showed that the grandparent-generation chickens, parents of broiler breeders, were imported from America with no disease record, which helped to exclude the factor of congenital transmission. In addition, the breeding conditions of the affected flocks presented no loopholes and the effects of other stimuli were extremely slight. Finally, when the source of infection could not be determined, we suspected the possible target-vaccine.

Nowadays, the quality of vaccines is becoming increasingly worthy of attention in poultry husbandry. For one thing, live-virus vaccines produced by using unauthentic SPF chickens or virus-free cells probably carried cell-free REV. For another, REV could be integrated into genome of DNA viruses such as MDV and fowlpox virus (FPV) etc. [5], [6], possibly leading to the contamination of the commercial vaccines. In the 1970s, the use of Marek’s disease (MD) vaccines accidentally contaminated with REV had been reported to induce a runting disease characterized by immunodepression and abnormal feathering in the vaccinated flocks in Japan and Australia [7]–[9]. Fadly and Witter [10] proved by in vivo and in vitro test that REV was a contaminant in a live virus fowl pox (FP) vaccine of poultry in 1997; Awad et al. [11] reported that one of the 30 detected FP vaccine samples was contaminated by REV in 2010. However, up to the present, there are few reports on Newcastle disease (ND) vaccines or infectious bronchitis (IB) vaccines of poultry contaminated with REV.

Here, we described an infection of REV in three broiler breeder flocks that had been vaccinated with commercial MD vaccine and ND+IB vaccine contaminated with REV. The data also demonstrated that the REV might be congenitally transmitted to 1 day old commercial chickens. The current paper emphasized a lack of quality control at the level of SPF production and vaccine production.

## Results

### Preparation of Probe

**Figure 1:**
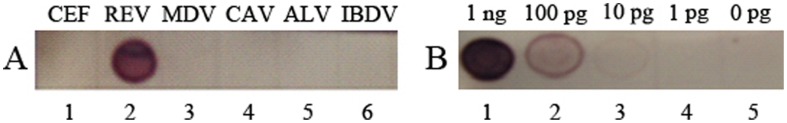
Examination of the specificity and sensitivity of REV env gene probe. (A) The specificity of REV env gene probe. Dot 1, DNA from CEF; dot 2, DNA from CEF inoculated with strain SNV of REV; dot 3, DNA from CEF inoculated with MDV; dot 4, DNA from CEF inoculated with CAV; dot 5, DNA from CEF inoculated with ALV; dot 6, DNA from CEF inoculated with IBDV. (B) The sensitivity of REV env gene probe. Dot 1 to 4, DNA from diluted PCR product of REV env gene, 1 ng, 100 pg, 10 pg, 1 pg, respectively; dot 5, no DNA.

REV env gene probe was labeled by DIG DNA labeling kit (described in Materials and Methods). The result of specificity examination showed that the probe, with good specificity, could only be reacted with cDNA of REV (Fig. 1A). And sensitivity examination showed that the probe with the final concentration of 50 ng/mL could be hybridized with serially diluted PCR product of REV env gene, and REV env gene was still sensitive to the probe when PCR product quantity reached 10 pg (color development 8 h) (Fig. 1B).

### Detection of REV Env Gene from Spleen Tissues

All 478 DNA samples from spleen tissues were detected by dot-blot hybridization followed by the statistics of the positive rates of REV-positive samples (Table 1). The results showed the REV-positive rates of the affected flocks were between 26.5% and 32.8%. Notably, 1 day old commercial chickens (bred by the affected breeder flocks) had markedly higher REV-positive rates which were between 43.5% and 48.3% than broiler breeders (*P*<0.05). The REV-positive rates of commercial chicken flocks were about 1.5–2 times as high as those of breeder flocks. No REV-positive chickens were detected in other flocks which were not affected by disease (data not shown). Because 1 day old commercial chickens had not yet received any vaccination, so the results suggest the infection of 1 day old commercial chickens was likely to be associated with congenital transmission.

**Table 1:** REV-positive rates of DNAs from spleen tissues in different generations (%).

Source of DNA	Age	REV-positive rate (n/N)^b^			
		Flock 2	Flock 3	Flock 5	Other flocks
Broiler breeders	25 weeks	28.3 (15/53)	32.8 (19/58)	26.5 (13/49)	0 (0/61)
Commercial chickens	1 day	43.5^a^ (27/62)	48.3^a^ (29/60)	47.1^a^ (33/70)	0 (0/65)

a vs. Broiler breeders, *P*<0.05.

b N, total number of samples; n, the number of positive samples.

### Detection of REV Env Gene from Cells Inoculated with Vaccines

**Figure 2:**
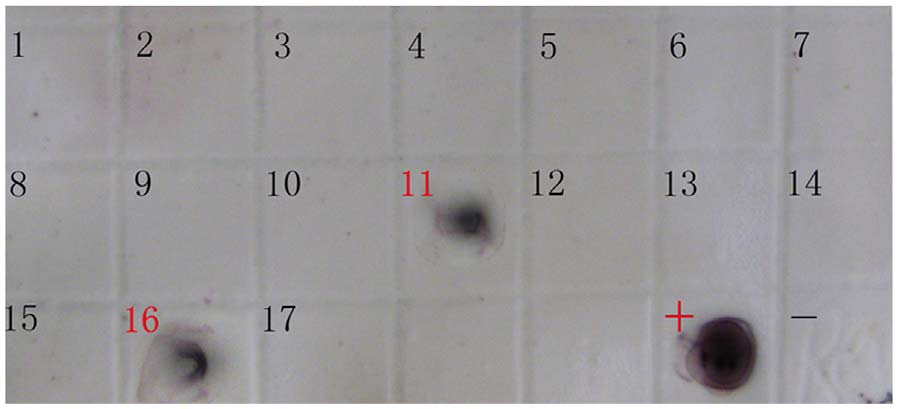
Detection of REV env gene from CEFs inoculated with vaccines. Dot 1–17: DNAs from CEFs respectively inoculated with ND-B1 vaccine, REO-1133 vaccine, FP vaccine, ND-LaSota vaccine, IBD-B87^1^ vaccine, IBD-B87^2^ vaccine, ND-CS2^1^ vaccine, ND-CS2^2^ vaccine, ND-LaSota+IBD-H52 vaccine, REO-1133 vaccine, MD-CVI988/Rispens vaccine, ND-CS2 vaccine, ILT-A96^1^ vaccine, ILT-A96^2^ vaccine, FP+AE-1143 vaccine, ND-LaSota+IB-H120 vaccine, and MG-F vaccine; dot + : DNA from CEF inoculated with strain SNV of REV; dot -: no DNA. The red numbers represent the positive results.

We collected 17 batches of live-virus vaccines used at different stages of the flocks (Table 2) and detected whether some of the vaccines were contaminated with REV. DNAs from CEFs inoculated with these vaccines were respectively detected by dot-blot hybridization with REV env gene probe. The results showed both DNAs from CEFs inoculated with MD-CVI988/Rispens vaccine (used at 1 day of age) and ND-LaSota+IB-H120 vaccine (used at 7 days of age) were REV-positive (Fig. 2). The outcome of investigation revealed both of these two vaccines were produced by the same manufacturer of China. Judging from the production methods, MD-CVI988/Rispens vaccine was produced by CEF, whereas ND-LaSota+IB-H120 vaccine was produced by allantoic fluid of SPF embryo, so the virus-infected CEF or SPF embryo probably was the source of contamination.

**Table 2:** List of vaccines used in the affected broiler breeder flocks.

Live-virus vaccine	Code name (virus abbreviations-strain)	Use of age (day)	Vaccination method	Country of origin
Marek ‘s disease vaccine	MD-CVI988/Rispens	1	Hypodermic injection	China
Newcastle disease vaccine	ND-B1	3	Intranasal and intraocular vaccination	USA
Avain viral arthritis vaccine	REO-1133	5	Hypodermic injection	Germany
Newcastle disease+Infectious bronchitis vaccine	ND-LaSota+IB-H120	7	Drinking water vaccination	China
Newcastle disease vaccine	ND-LaSota	12	Intranasal and intraocular vaccination	China
Infectious bursal disease vaccine	IBD-B87^1^	12	Oral vaccination	Germany
Infectious bursal disease vaccine	IBD-B87^2^	20	Drinking water vaccination	Germany
Fowl pox vaccine	FP(quail attenuated strain)	24	Stab vaccination	China
Infectious laryngotracheitis vaccine	ILT-A96^1^	30	Intraocular and anal vaccination	Indonesia
Newcastle disease+Infectious bronchitis vaccine	ND-LaSota+IBD-H52	35	Intranasal and intraocular vaccination	China
Chicken viral arthritis vaccine	REO-1133	50	Hypodermic injection	Germany
Newcastle disease vaccine	ND-CS2	60	Drinking water vaccination	China
Infectious laryngotracheitis vaccine	ILT-A96^2^	85	Intraocular and anal vaccination	Indonesia
Fowl pox+Avian encephalomyelitis vaccine	FP+AE-1143	100	Stab vaccination	USA
Mycoplasma gallisepticum vaccine	MG-F	100	Intraocular vaccination	China
Newcastle disease vaccine	ND-CS2^1^	120	Hypodermic injection	China
Newcastle disease vaccine	ND-CS2^2^	160	Hypodermic injection	China

The numeric superscripts “1”and”2” are to differentiate two of the same code names.

### Verifying the Accuracy of Dot-blot Hybridization

**Figure 3:**
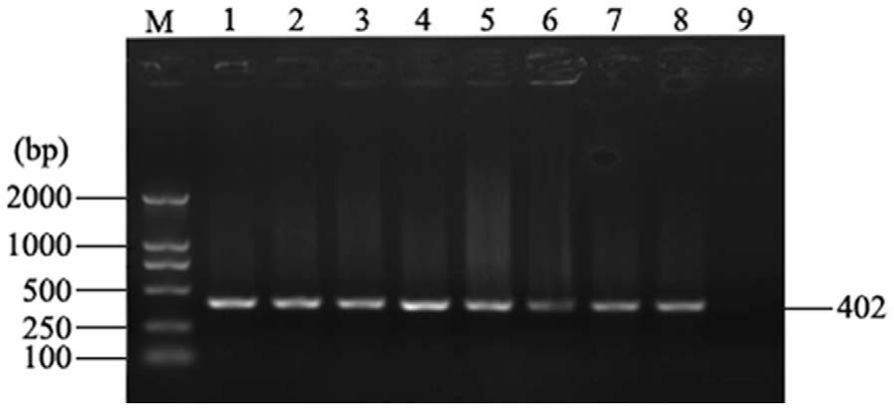
Verifying the accuracy of dot-blot hybridization. Lane M, DL 2000 DNA marker; lane 1 to 3, DNAs from spleens of the diseased broiler breeders; lane 4 and 5, DNAs from spleens of the 1 day old commercial chickens; lane 6, DNA from CEF inoculated with MD-CVI988/Rispens vaccine; lane 7, DNA from CEF inoculated with ND-LaSota+IB-H120 vaccine; lane 8, DNA from CEF inoculated with strain SNV of REV; lane 9, no template.

To verify the accuracy of dot-blot hybridization, we adopted a nested PCR assay. The REV-positive DNA samples were randomly selected as the templates. Finally 402 bp PCR products were observed by agarose gel electrophoresis (Fig. 3). Fig. 3 showed part of the testing results. All the testing results of the randomly selected templates were consistent with those of dot-blot hybridization.

### Cell Culture Isolation of REV from Selected Vaccines

**Figure 4:**
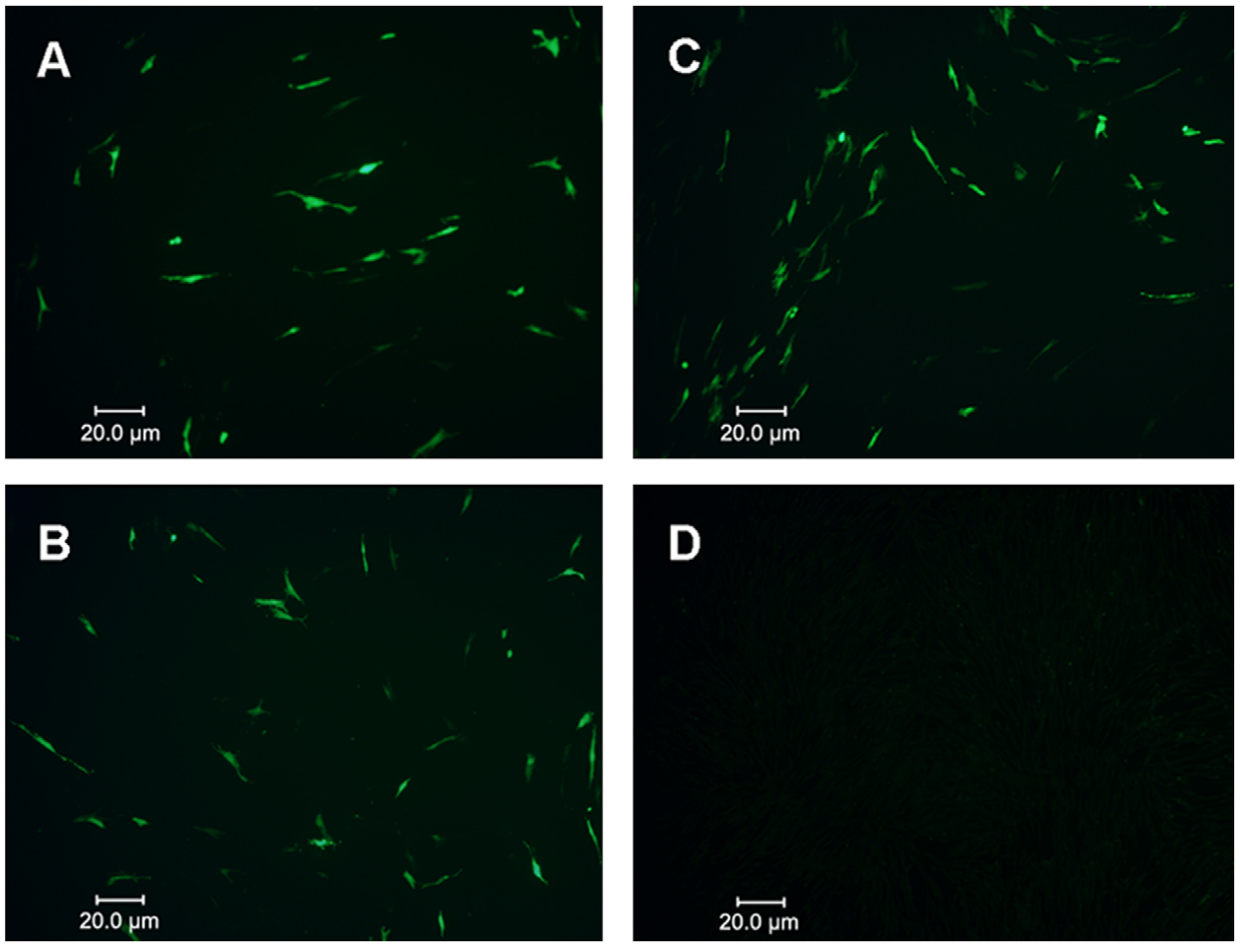
Cell culture isolation of REV from the selected vaccines. (A) CEF inoculated with dilution of MD-CVI988/Rispens vaccine. (B) CEF inoculated with dilution of ND-LaSota+IB-H120 vaccine. (C) CEF inoculated with dilution of REV-SNV strain. (D) Normal CEF as negative control.

To further detect whether the contaminated vaccines carried cell-free REV, the diluents of MD-CVI988/Rispens vaccine and ND-LaSota+IB-H120 vaccine were inoculated into CEFs and then detected by indirect immunofluorescence assay (IFA). The results showed that CEFs inoculated with these two types of vaccines both contained REV-positive cells. It suggested that both vaccines were contaminated cell-free REV (Fig. 4A, Fig. 4B). However, the REV-infected cells in Fig. 4A or Fig. 4B were less than REV-SNV-infected ones (Fig. 4C), which was probably due to a low concentration of the contaminant REV in both vaccines.

## Discussion

REV infection in chickens generally results in decrease of production performance and decline of immune function, and is likely to cause vaccination failure [12], [13]. REV is spread mainly through contact with infected poultry such as chickens, turkeys and ducks, and through insects as well [14], [15]. Congenital transmission of REV in chickens and turkeys had already been reported, whereas cases in 1980–1990s were mostly proved while the reports were rare in the last 10 years [16], [17]. In addition, due to a long latent period and the polyinfection with other oncogenic virus such as MDV, ALV etc., the symptoms caused by REV were usually disguised [18], [19], and therefore the examples of the typical congenital transmission of REV were not often seen.

Our group had been all along doing survey on epidemiology of REV infection in chickens. It had been found that if the commercial chickens were detected with REV-positive, then the homologous broiler breeders tended to be REV-positive, and commercial chickens generally had a higher infection rate than broiler breeders did. In the present study, a suspected REV-infected broiler breeder company of Shandong Province was selected. DNA samples from the diseased broiler breeders in the affected flocks were detected by dot-blot hybridization. The results showed that the diseased broiler breeders of the three affected flocks were positively infected with REV. Further, a follow-up investigation showed that the REV-positive rate of the 1 day old commercial chickens had reached 48.3%, 1.5–2 times as much as that of the broiler breeders. Although the REV-positive rate of 1 day old commercial chickens could not represent the real value at 25 weeks old, the results of the present study suggested that REV infecting the affected broiler breeder flocks was very likely to have been transmitted to the next generation by congenital transmission.

In the face of the affected flocks, we could not determine the exact source of infection. But compared with other healthy flocks, it was discovered that the vaccines were not completely the same. Subsequently, we detected REV env genes from CEFs inoculated with the 17 batches of live-virus vaccines used in the affected flocks by dot-blot hybridization, and found MD-CVI988/Rispens vaccine and ND-LaSota+IB-H120 vaccine were REV-positive. IFA further proved that both batches of vaccines were indeed contaminated with cell-free REV. Contamination of Newcastle disease vaccine or infectious bronchitis vaccine of poultry with REV has not been previously reported. Through investigation, both contaminated vaccines used on the affected flocks were produced by the same manufacturer, while the vaccines of the unaffected flocks were purchased from different ones. The results suggested that the infection of REV among broiler breeders was most probably caused by the contaminated MD-CVI988/Rispens vaccines and ND-LaSota+IB-H120 vaccines. These vaccines were commonly administrated to broiler breeders at early breeding stage, and therefore these vaccines contaminated with REV would very likely to cause REV-infection of broiler breeders. In addition, these unqualified vaccines might not only be the sources of infection of broiler breeders, but also congenitally transmitted the virus to commercial chickens.

To date some proofs revealed that, conventional live-virus vaccines produced by SPF embryos or CEFs were often contaminated by cell-free REV [7], [8], [9], [10], [20]. In addition, REV could also be integrated into genomes of DNA viruses such as MDV or FPV [5], [6], and thereby possibly led to the contamination of commercialized DNA vaccines. Generally speaking, it’s not often seen that intact REV gene was inserted into the genome of vaccine strain [21]. More probably, it’s the administration of live-virus vaccines produced by unauthentic SPF embryos or virus-free cells to some breeder flocks that resulted in REV-infection. And it was likely to be a major cause of the fact that the infection rate of immunosuppressive virus in China was higher than that in other major poultry-raising countries of the world. Therefore, improvement of controls to prevent contamination before and during production of commercial poultry vaccine must be one of the necessary measures in ensuring the biosafety [20].

REV usually exists within cells in the form of provirus with latent infectivity. Its RNA genome generally transforms to cDNA within nucleus through reverse transcription and is integrated into DNA of cells, so molecular biology techniques are commonly used in diagnosis of REV [22], [23]. Dot-blot hybridization has sensitive specificity and it is suitable for rapid handling of a large number of specimens [5]. In addition, the contaminated vaccines had extremely low REV concentrations, resulting in little amount of cDNA from CEF infected with REV, hard for the conventional RT-PCR to detect. So we performed nested PCR to verify the results of dot-blot hybridization [24]. Furthermore, we located the virus directly in cells with IFA. All of these methods ensured the accuracy of this study.

## Materials and Methods

### Ethics Statement

All animals were handled in strict accordance with good animal practice as defined by Shandong Institute of Animal Husbandry and Veterinary, China, and the whole procedure for collection of the tissue samples was carried out in strict accordance with the protocol approved by the Animal Welfare Committee of Shandong Agricultural University.

### Cell Culture

Chicken embryo fibroblasts (CEF; derived from 9 days old SPF embryos) monolayers were maintained in the exponential growth phase in Dulbecco’s modified Eagle’s medium (Gibco, USA) supplemented with 10% fetal bovine serum (Hyclone, USA), 100 units/mL penicillin, and 0.1% (w/v) streptomycin.

### DNA Isolation

Firstly, spleen tissues of the diseased broiler breeders (25 weeks old) were collected, 221 in all. Subsequently, the spleen tissues of 1 day old commercial chickens bred by the affected breeder flocks (not received any vaccination; provided by Shandong Lvyuan Broiler Sales Co., Ltd.) were collected, 257 in all. Genome DNAs were extracted with DNA extraction kit (Roche, Germany).

Seventeen batches of freeze-dried vaccines used in the affected breeder flocks were provided by the chicken farm (Table 1). Three bottles were sampled randomly from each batch of vaccines. Each bottle of vaccine was diluted with 5 mL of 1×PBS (phosphate-buffered saline). After filtering, 1 mL of diluent was inoculated into the 70% CEF monolayer. After 72 h of culture, supernatant was inoculated into a new CEF monolayer. After another 72 h of culture, cells were washed and collected. Genome DNAs of CEFs were extracted with DNA extraction kit (Roche, Germany).

### Preparation of Probe

Env-F and env-R were designed and synthesized as the primers of PCR (Table 3), pB101 plasmid containing REV env gene cDNA clone (provided by avian disease and oncology laboratory of Shandong agricultural university) was served as template to amplify REV env gene (438 bp). After identification of the PCR product (Shanghai Sangon Company), REV env gene was labeled with DIG (digoxigenin) DNA labeling and detection kit (Roche, Germany). The hybridization and detection were conducted according to the instruction of the manufacturer.

**Table 3:** List of primers used for amplification of REV env gene.

Primer name^a^	Sequence (5′–3′)	Position in REV genome	Target fragment	Fragment size (bp)
env-F	AGCTAGGCTCGTATGAA	6504–6520	env gene	438
env-R	TATTGACCAGGTGGGTTG	6940–6923		
env-nested-F	ATGAAGACGGGCCTAA	6515–6531	env gene	402
env-nested-R	AAAGGGGAGGCTAAGA	6916–6910		

a F, forward; R, reverse.

To determine whether the labeled probe reacted with other DNAs/cDNAs of immunosuppressive virus, we performed dot-blot hybridization using positive DNAs/cDNAs (provided by avian disease and oncology laboratory of Shandong agricultural university) of REV (SNV strain), MDV (GX0101 strain), ALV-J (NX0101 strain), chicken infectious anemia virus (CIAV, TJBD40 strain), and infectious bursal disease virus (IBDV, GX8/99 strain) to examine the specificity of the probe. Then quantitative PCR product of REV env gene was serially diluted 10-fold in ddH2O, which was followed by the performance of dot-blot hybridization to detected the sensitivity of the labeled probe.

### Dot-blot Hybridization

Probe, positive DNAs/cDNAs, or DNA samples from tissues and CEFs were denatured at 94°C for 7 min, and then and then went through an immediate 5 min ice bath. 2 µL of denatured DNA was spotted onto the Nitrocellulose membrane (NC membrane, Pall, Mexico), and was baked for 2 h at 80°C to fix DNA. Then Dot blot hybridization was conducted according to the protocol of Ni and Cui [14]. Finally, NC membrane was processed for color development with BCIP/NBT Alkaline Phosphatase Color Development Kit (Sigma, USA).

### Nested PCR

Randomly selected DNA samples proved to be positive by dot-blot hybridization from different tissues and CEFs were verified by nested PCR, and two pairs of primers were designed and synthesized based on provirus genome cDNA sequence of REV env gene (SNV strain; GenBank accession number: DQ003591) (Table 3).

The first round of amplification was in accordance with the following reaction system: 0.2 µL of template DNA, 5 pmol of primers (env-F and env-R), 4 nmol of dNTPs, 2 µL 10×PCR of buffer, 1U of Taq DNA polymerase (TaKaRa, Japan), and 20 µL of total system. Reaction condition: 95°C 4 min, 95°C 30 s, 54°C 30 s, 72°C 30 s, 28 cycles, and finally 72°C 10 min. In the second round of amplification, the template was the PCR product of the first round, and the primers were env-nested-F and env-nested-R. The reaction system and reaction condition were the same as those of the first round. Finally, Nested PCR product was subjected to 1% agarose gel electrophoresis.

### IFA

Each bottle of vaccine was diluted with 10 mL of 1×PBS, 1 mL of diluent was inoculated into the 70% CEF monolayer. The diluents of all vaccines were inoculated on the 96-well plate, 3 wells for each vaccine sample. After 72 h of culture, cells infected with REV were located by IFA [19]. In this assay, cells infected with SNV strain of REV were used as positive control, while the normal cells as negative control. The anti-REV monoclonal antibody 11B118 was provided by avian disease and oncology laboratory of Shandong agricultural university and FITC-conjugated goat anti-chicken IgG antibody was purchased from Sigma, USA.

### Statistical Analysis

SPSS V14.0 software was used for the statistical analysis. The level of significance was *P*<0.05.
